# Societal dangers of migrant crisis narratives with a special focus on Belarussian and Ukrainian borders with Poland

**DOI:** 10.3389/fsoc.2022.1084732

**Published:** 2023-01-18

**Authors:** Izabela Grabowska

**Affiliations:** Department of Economics, Center for Research on Social Change and Human Mobility (CRASH), Kozminski University, Warsaw, Poland

**Keywords:** migrant crisis narrative, societal danger, Belarussian-Polish border, Ukrainian-Polish border, societal fatigue, othering, political functionality

## Abstract

Society in the 21st century has experienced a variety of crises, from the fiscal crisis and the migration crisis to the pandemic and the inflation crisis. This paper aims to explore societal dangers of migrant crises narratives. This paper forms part of the Horizon 2020 MIMY research projects with an expert stakeholder Delphi study from seven European countries: Germany, Italy, Poland, Romania, Luxembourg, Sweden and the UK. It takes also into account contextual international and national public opinion surveys. We formulated a number of societal dangers related to the migrant crisis narrative, which are not sharp and exclusive but invite further consideration: (1) *Societal fatigue*, which relates to a rapid change in societal moods, usually from a positive to a negative attitude toward migrants, but above all this danger is connected with an aid burnout in a civil society; (2) *Othering*, which includes normativity, the labeling of migrants, double or multiple standards in the treatment of migrants and refugees from various origins; the societal danger of othering contributes to societal divisions, polarizations, tensions and conflicts based on ethnicity, religion, race and gender; (3) *Political functionality*, whereby migration as a political construct serves as a “whipping boy” for politicians to divert public opinion from recurrent problems; it also involves the creation of piecemeal, reactionary, ad hoc public policies, and the overuse of a protocol of a state of emergency in order to bring about a centralization of political power.

## 1. Introduction

The migration crisis is a big topic, and this is a short paper. In our analysis, we attempt to shake a few societal dangers of using the term of “migration crisis narrative” in a society. By identifying the dangers—the list is not complete and open-ended—we wish to draw attention to some areas of public policy and the gaps within them. Society in the twenty-first century has been generally marred by crisis in one form or another (cf. Walby, 2015), from the fiscal crisis, through the migration crisis and the pandemic crisis to the inflation crisis. The word “crisis” derives from Ancient Greek, where it means a power of distinguishing or separating, decision, choice, election, judgment, dispute. Economists relate the word crisis to a decisive moment for economic transformations. Sociologists relate it to social change. Migration scholars consider all of the above, and ask why human geographical mobility is so easily paired with crises (cf. Bello, 2022a,b).

The term “migration crisis” problematizes migrants to a receiving society. The question, however, is: who or what exactly is in crisis here? The migrants themselves, or the political system or the society to which they migrate? The term alludes to the undesirability of a situation or a process. The “migration crisis” narrative helps keep society at a standstill, bounded, territorially bordered. The narrative around migration crisis engages individuals, groups and institutions. It is important to establish whether crisis is treated as real in a society or as a socially constructed narrative, or whether it is socially constructed by a narrative and therefore becomes real (Walby, 2022). “The analysis of a crisis involves both aspects: it is real and it is socially interpreted, which has effects. The interpretation of a crisis as permitting or requiring a state of emergency to be declared produces a centralization of political power that can have consequences” (Walby, 2022, p. 4). Crises represent moments of uncertainty and confusion in which civil organizations emerge to diagnose what has gone wrong and take action. This involves making sense of a given crisis by reducing its present complexities to identifiable causes and consequences. The literature brings complex problematizations of the crisis (Greussing and Boomgaarden, 2017), and links the migrant crisis with other recent and ongoing crises in Europe—the financial crisis of 2008, the Eurozone crisis and the crisis of security (cf. Falkner, 2016; Seabrooke and Tsingou, 2019), the pandemic crisis of 2020–2021 and the inflation crisis of 2022. It also highlights how the migrant crisis intersects with other crises. Migrant issues and especially immigration and integration policies are perfect lens to observe a production of migrant crisis narratives (cf. Boswell et al., 2011). Migration is also such a social phenomenon to be a vehicle to inspire political mobilization and to create a reference for a political conflict.

Policy narratives have their own dynamic which distinguishes them from other public debates and links them with social claims about values or political interests (Boswell et al., 2011). The credibility of policy narratives depends on sources of knowledge and their reliability as based on either a personal experience, a personal perception, a knowledge from media news or knowledge based on research evidence (Boswell et al., 2011). Narratives are shaped by political approach and their traditions in a society, and are influenced by competing actors to “frame” issues according to their political functionality. In order to have its social power, policy narratives need to be compelling, quite comprehensive and coherent. Migrant policy narratives are the space to observe the above mentioned aspects (Boswell et al., 2011).

We formulate four guiding research questions of this article: (1) What is a difference between a policy narrative and an individual narrative? At which level of analysis the policy narrative connected to migration is located?; (2) What are the factors stimulating migrant crisis narratives?; (3) What new arguments an expansion of migrant crisis narratives to Central and Eastern Europe (CEE) brings, especially to its Belarussian-Polish and Ukrainian-Polish borders? (4) What are societal dangers of overusing and not-using migrant crisis narratives in specific circumstances? The key aim of this article is to explore social factors that stimulate the “migrant crisis” narrative and societal dangers caused by migrant crisis narratives. The paper is divided into five sections: introduction, theory, methodology, findings, and concluding discussion, including some thoughts on implications for social theory and policy.

## 2. A conceptual approach to the migrant crisis narrative

This section locates our discussion and research at the intersecting point of three bodies of literature: the general sociological meaning of crisis, the migrant crisis through the lens of migration studies, and the societal narrative from the perspective of both general sociology and migration studies. This literature review builds a framework for the analysis of the societal dangers of the migrant crisis narrative.

The concept of a migrant crisis can be understood only within a society. Whether it has a transformative power depends on its structures (cf. Walby, 2022), but also timing and space. The migration crisis fits the definition of crisis offered by Walby (2015) as “an event that has the potential to cause a large detrimental change to the social system and in which there is lack of proportionality between cause and consequence” (Walby, 2015, p. 1). However, there are two aspects that require further discussion. The interpretation of migrant crisis as an event may be correct, but how should we interpret its “ongoing-ness”? After all in Winter 2022, (1) people have been fleeing *via* the Mediterranean Sea since the summer of 2015, and later also through English Channel, and are still doing so; (2) there are still people in forests of Belarus on the Belarussian-Polish border, who have been there since early Autumn 2021; and (3) Ukrainian refugees have been fleeing massively the Russian war since February 2022. The other aspect relates to the fact that human migrants are involved and one cannot talk about a “migrant crisis above our heads”. The definition offered by Walby, however, as “an event in a short period of time and a longer period of consequences that cascades in non-linear form” helps make sense of developments related to migrant crises (Walby, 2022, p. 14).

In the field of migration studies, various terms are used for the migrant crisis: the “refugee crisis” (Khiabany, 2016, p. 755), the “migrant crisis”, the “refugee and migrant crisis” (Karolewski and Benedikter, 2017, p. 294) (cf. Kushnir et al., 2020). The term “migration crisis” is not new or isolated in the literature as a phenomenon or assigned to a particular event (cf. Weiner, 1995). “It is rather one among a series of scattered inflamed reactions to recurrent massive movements of people. (…) global migration crises [are] socially constructed scattered inflamed reactions that have been happening since the end of Cold War, as a consequence of forced movements of people that a variety of conflicts and instabilities have produced across the planet” (Bello, 2022a,b, p. 1327). As Kushnir et al. (2020) discuss, mixed migration flows and types of migration become conflated and misinterpreted, which exacerbates the migration crisis narrative. We understand in this article the “migrant crisis” as being located at the intersection of various “crises”.

Crawley (2016) analyses the policy response to migration crisis, mostly that of 2015, and states that it does not reflect the numbers but differences between states, the EU and other parts in relation to their perception of migration. He means the unwillingness and inability of politicians and policymakers to use extensive evidence on migration dynamics and to apply political and economic resources to address the consequences of conflicts and economic underdevelopment in migrants' areas of origin (Crawley, 2016, p. 14).

Societal narratives tell us about societies, their past and their imagined future. Societal narratives are usually located at meso levels—between local narratives produced by individuals and organizations, national/policy narratives produced by ruling governments, and the global metanarratives (Lyotard, 1979) produced by international organizations and global media. Societal narratives reflect and affect the properties of the societies where they emerge (cf. Corvellec and Hultman, 2012). In order to understand the meso level of narratives, more deliberation is needed in this article.

As Bello says (2022b, p. 1445–1446), narratives can be openly rejected by some, but they do not need to be accepted by the others to have an effect. The difference between an act of speech and a narrative is that first the audience needs to accept a message formulated by the sender (e.g., the government in power) then it is a spill-over into a narrative. From a postmodern perspective, the narratives represent “true knowledge” that cannot be challenged by the audience (Lyotard, 1979), but needs to be “resisted” with alternative narratives often offered by civil society.

Boswell (2011) and Boswell et al. (2011) talk about policy narratives and embed both crises and their narrative(s) connected to migration into a migration policy-making. Migration issues compete with other policy narratives and somehow often, especially when radical but systematically and coherently repeated and connected with other policy areas are appealing to an audience. “Many aspects of migration control can be characterized as areas of risk, with policy-makers forced to make decisions with potentially beneficial or harmful consequences under conditions of great uncertainty” (Boswell et al., 2011, p. 3). Policy narratives can spread across sectors, regions and even countries.

For the purpose of this article, we therefore define the migrant crisis narrative as a narrative located in-between the level of policy-makers (cf. Boswell et al., 2011), associated here with a ruling government and the local level of individuals and organizations acting systematically on the ground and self-reporting their work, primarily through social media, and thereby usually offering an alternative, bottom-up narrative in reaction to the top-down narrative spread by right-wing politicians, who in some countries are the ruling governments. It is therefore worth considering the extent to which these crisis narratives are top-down—e.g., from the government and policy makers, the media etc.—or rather bottom-up, i.e., emanating from the populace?

## 3. Methodology

To address the societal dangers of migrant crisis narratives requires both theoretical thought and some degree of empirical exploration. The data informing this article was collected through the Delphi stakeholder study's results conducted in the international Horizon 2020 MIMY international research project^1^ in two waves addressing issues of migration and integration policies, decisions, distribution of power and implementations, as well as through the review of both international and national public opinion survey results and societal sentiment analyses on social media, mostly Twitter. We need to make a disclaimer here that we did not study, however, in this article the impact of traditional media and social media on migrant crisis narratives which might be a separate topic for a new article. An earlier evidence shows, however, a complementary not competing effects between the two: more general and neutral picture of refugees was presented by traditional media and more individualized, empathic picture of refugees was presented by social media (cf. Nerghes and Lee, 2019). There is a lack of findings however about the role of public media turned into propaganda media like in Poland and Hungary for instance and their role in producing migrant and refugee pictures leading to crisis narratives as juxtaposed to social media in these countries.

For our primary data collection we used an international longitudinal stakeholder survey developed in H2020 MIMY research project. Our stakeholder survey was conducted in a mode of a Delphi Study. A Delphi study is literally a virtual panel of stakeholders who come virtually together without knowing their identities to arrive at a collective answer to a challenging question. Thus, a Delphi study could be considered a type of virtual meeting or as a collective consensus-seeking approach (cf. Linstone and Turoff, 1975). The Delphi survey is a tool designed to systematically collect information from a group of stakeholders in a way that decreases individual bias and reduces uncertainty about the future (Dalkey and Helmer, 1963). We used a mixed-method approach in one, combined research tool of a Delphi Study (cf. Linstone and Turoff, 1975). It was combined of standardized survey questions with numerical scales capturing the earlier findings of the H2020 MIMY research project and open qualitative questions where answers can be hand-written.

People observing and analyzing migrant integration over years, designing migration policies or directly working with migrants can provide valuable intuition and knowledge about integration patterns. Stakeholders—especially when consulted in groups—can help resolve conflicting knowledge and enhance awareness about uncertainties, which ideally leads to a situation in which groups perform better than its single best member (Rowe et al., 1991).

We received ethical approval from the Research Ethics Committee of Kozminski University in Warsaw who was responsible for this task in the international consortium of H2020 MIMY to coordinate and conduct the international survey with stakeholders of migration and integration issues from partner countries who operate both locally and nationally which helped us gather diverse points of view. The sample was purposive and based on previous contacts and participation of these stakeholders in the international H2020 MIMY, assembled in a so called MIMY Stakeholder Platform. The platform was built earlier than the survey was launched and aimed to create a sense of connection to the project throughout its duration, for stakeholders so that they become collaborators in the process. The stakeholder platform embedded into local and national contexts of partner countries provided the contacts for engagement at the local and national levels within our stakeholder study. Every consortium partner was responsible for recruiting and maintaining contacts with stakeholders from their country. It explained to actors being recruited into the platform to engage in the whole research project.

Our Delphi Study is distinct in four ways: (1) it involved stakeholders already active in the project on other occasions at Stakeholder Platform; (2) research tools (questionnaires) were consulted with migrant organizations, including youth migrant organizations; (3) it involved both policy makers, policy users and observers.

We collected data from stakeholders-participants from seven European countries in two waves of the study: Italy, Luxemburg, Poland, Romania, Sweden and the UK. Altogether, the first stage of the study involved 114 stakeholders^2^; the second stage, in which we also received qualitative answers, involved longitudinally 45 stakeholders. The first wave was conducted anonymously from December 2021 to March 2022 and the second wave was also conducted anonymously from June to September 2022. In the second wave we showed the results to our stakeholders from the first wave so they could anonymously experience the opinions of the others and participate in a learning process. The study was conducted with the help of internet survey (*Computer Assisted Web Interview*, CAWI) with some open questions. Both questionnaires were coded in JotForm in six languages and the data was gathered in one excel database. This made it possible to send the online survey questionnaire links to the stakeholders in their chosen language of communication. Each country partner in the consortium of H2020 MIMY research project was responsible for the management and maintenance of the contact with stakeholders in their country. In general, project partners involved stakeholders defining themselves (multiple answers) as advocacy (*n* = 40); policy users (*n* = 29); migrant organizations, including young migrant organizations (*n* = 25); lobbying (*n* = 18); both policy maker and policy user (*n* = 17) and policy maker only (*n* = 9). Some of survey participants perform multiple roles. A *policy maker* was defined as a person who is responsible for policy strategy, framework, and a design of instruments. A *policy user* was defined as a person who is responsible for putting the policy into practice (e.g., street worker; counselor; social workers; family assistant; career advisor; and migrant club animator), and applies it into a practical context. *Instruments* linked to migrants are understood here as practices, tools which can be used to overcome challenges and to achieve aims.

The majority of the stakeholders who took part in this study were women (*n* = 72). Thirty-five men took part in this two-wave survey and seven people stated that they did not want to share their gender. We also asked about the migration backgrounds of the stakeholders themselves, considering that they work for and with migrants; 23 people among our stakeholders stated that they have a migration background, 83 said no migration background and 6 did not want to share. The average age of our respondents was around 40. This study by the H2020 MIMY research project aimed to, in a dialogue and in one longitudinal study, juxtapose various perspectives from policy makers and policy users, especially between makers at the national level and users at the local level in order to work out issues for migration/integration policies.

## 4. Societal dangers of migrant crisis narrative and its factors

Before we attempt to understand the societal dangers of the migrant crisis narrative, it is important to explore the factors that stimulate and facilitate the migrant crisis narrative in the literature as well as in the findings of the stakeholder study of H2020 MIMY research project.

A multitude of factors shape the migration crisis narrative. Among the factors stimulating the migrant crisis narrative is the so-called “new order of social uncertainties” as elaborated by Appadurai (2006). This new order is characterized by: (1) uncertainties about stability, existence, state goods and their redistribution; (2) a loose or non-existent connection with Weberian predictable bureaucratic and legalized procedures: (3) problems with predictions—unexpected occurrences with global impact (e.g., pandemics and war); (4) a lack of security of health and sanitation (especially during COVID-19 pandemics); and (5) a lack of affordable housing for the under-waged, young, lower middle class. These new forms of uncertainty create intolerable anxiety and uncertainty (Appadurai, 2006). Especially when such uncertainty is allied with other social forces, such as the growing disregard for inequalities, the disregard of citizen protests by the state, and *ad hoc*, piecemeal actions accelerated by a crisis narrative. Harris (2021) also points out a number of factors which may stimulate and therefore facilitate a migrant crisis narrative, which are in line with Appadurai (2006): (1) growing economic inequalities exacerbated by pandemics; (2) polarized political climate: the tightening of borders, restrictive policies, visa complexity; and (3) uncertainties about stability, existence and state goods and their redistribution.

In our international stakeholder study under H2020 MIMY research project, we asked our respondents about the extent to which they agree that the following factors (from a prescribed list developed on the basis of earlier findings of the project) might constrain policy regarding migrants and their prospective integration. A lack of political will was chosen by all stakeholders from the studied countries, especially Swedish stakeholders (mean 4.65/5.00) and Polish stakeholders (4.53/5.00). Italian, Romanian and British stakeholders also strongly confirmed this (all 4.47/5.00), with respondents from Luxembourg (4.38/5.00) and Germany (4.33/5.00) the least emphatic. Lack of knowledge as a factor constraining migration and integration policy was mostly chosen by Luxembourgian (mean 4.50/5.00) stakeholders and German stakeholders (also 4.50/5.00), less so but still to a great extent by Swedish (4.41/5.00) and Romanian stakeholders (also 4.41/5.00), followed by the Italian (4.35/5.00) and British stakeholders (4.33/5.00) and finally the Polish stakeholders (4.24/5.00), though even this lowest score is still high.

When we asked our stakeholders about the factors impacting the shape of a policy concerning migrants (also from a prescribed list), they mostly chose: (1) populist government in power (mean 4.55/5.00); (2) the influence of all kinds of media, including social media (mean 4.13/5.00); (3) the financial crisis (4.00/5.00); (4) the funding of NGOs and limited or lack of funding (3.95/5.00); (5) economic slowdown (3.90/5.00); (6) the situation in countries of origin (3.70/5.00); and (6) the pandemic (3.45/5.00).

The following section presents the second stage of our analysis, in which we formulate a number of societal dangers of the overuse or, conversely, the purposeful non-use of the term “migration crisis” in the public space: (1) societal fatigue; (2) othering; and (3) political functionality. These dangers can operate as inhibitors because they can reduce or suppress the actions of actors in the society. The three societal dangers presented below are not mutually exclusive by any means. They relate to each other and sometimes overlap.

### 4.1. Danger 1. Societal fatigue

The repeated use of the term “migrant crisis” over a period of time brings internalization of social fatigue in a society. Social fatigue means that members of a society run out of energy to spend on incoming forced, war migrants^3^. Social fatigue caused by the “migrant crisis” narrative leaves society overstimulated, stressed, tired, anxious, negative, and under pressure in given social settings. Social fatigue also results in indifference to social problems.

Baláž et al. (2021, p. 5) found that population-related factors such as stock of foreign-born population and a sudden increase in migration flows, types of settlement and sociodemographic variables impact significantly on long-term attitudes toward immigrants (2020, p. 11). According to BaláŽ et al., the 2015 migration crisis has a stronger impact on feelings toward immigrants than terrorist attacks. This effect is especially strong in post-communist countries. The radical increases in “very negative” statements correspond with the large number of early arrivals of migrants and refugees *via* the Balkan route. Very negative attitudes toward migrants from outside the EU have been growing substantially in post-communist countries since November 2015, usually along the line of the narratives of national governments. The percentage of “very negative” feelings slightly decrease over time but stay high in some EU countries at the end of 2018. Countries located on the Balkan route such as Greece, Romania and Bulgaria had high numbers of transit and, or staying migrants, but expose lower level of “very negative” attitudes toward non-EU immigrants compared with post-communist countries outside the Balkan route such as Czechia, Slovakia, Latvia and Estonia (Baláž et al., 2021, p. 12).

Krzyżanowski (2018), Krzyżanowska and Krzyżanowski (2018), and Żuk and Żuk (2018b) showed that the societies of Central and Eastern European countries presented a more racist attitude during the “2015 crisis” than Western European societies. Why? There is not a straightforward explanation. There might be still a cultural and political gap that divides the European Continent—a Berlin Wall remaining in people's heads. But is it that simple? Already in 2009 The Pew Global Attitudes Survey looked at differences between Eastern and Western societies of Europe. It found that Central and Eastern Europeans were less accepting that “a good thing for any society to be made up of people from different races, religions and cultures”. Thirty percent of Hungarians and 22 percent of Poles did not favor that diversity was a good thing, compared with one in 10 of the French and 13 percent of British and Germans. When asked about specific ethnic groups, the picture blurred. In Central and Eastern Europe, anti-Semitism was still prevalent known as a prejudice without factual presence of Jews, while in Western Europe people show negativity to Muslims. The Pew survey found that nearly 30 percent of Poles and Hungarians had negative attitudes to Jews while nearly 30 percent of British and nearly 70 percent of Italians had a negative view of Muslims, while 30 percent of Germans did not like Turks. Western Europeans may potentially look more tolerant when talking in the abstract, political correctness's terms, but are also somehow intolerant with attitudes addressing a specific ethnic group. (cf. Pew Global Attitudes Project, 2009).

Coletto et al. (2017) analyzed sentiments toward the refugee crisis in 2015 using Big Data from Twitter. They collected tweets posted in English from mid-August to mid-September 2015. Their dataset comprised ~1.2 million tweets from 47,824 users, excluding bots. They classified each user on a binary basis, i.e., as having either positive or negative sentiments toward the refugee crisis. The study revealed that at the beginning Europeans mostly expressed positive sentiments toward the refugees.

Righi et al. (2021) collected around 2,400 Italian-language^4^ tweets per day in the period of January 2015 to October 2018 and classified their mood using unsupervised sentiment analysis to derive an index of migration mood (DIV) based on the ratio between the number of positive tweets to the sum of positive and negative tweets. Their analysis shows that the mood toward migration seemed to move from initially positive to negative during the summer 2016 crisis, when the arrivals of migrants consistently increased, with the negative sentiment deepening after March 2018 (cf. Bosco et al., 2022). Negative attitudes, especially as a reversal of previously positive attitudes, can be also a proxy of a societal fatigue.

The other example concerns the hostile narrative of the Polish government relating to the situation on the Belarussian-Polish border in Autumn 2021 as regards the inflow of migrants and refugees from the Middle East facilitated by Lukashenka's regime which was reflected in the public opinion surveys and also by the manner in which survey questions were formulated. For instance, the survey conducted by SW Research for the Polish center-right daily newspaper *Rzeczpospolita* on November 9th−10th, 2021 asked respondents whether, in their opinion, the situation on the Polish-Belarusian border threatened the security of Poland. Nearly 70 percent answered “yes”, 15 percent said “no” and 15 percent had “no opinion”. Later the same month, another survey was conducted by the same company and it was directly commented as “the crisis” on the Polish-Belarussian borderland in the reference to the State of Emergency introduced by the Polish government as a way to exhibit a centralization of the political power (cf. Walby, 2022). At this point, 63 percent respondents thought that the situation on the border was a threat to the security of Poland and nearly 68 percent said that it was a threat to the entire European continent. However, half of the respondents (mostly with political affiliation to opposition, from big cities and higher educated) noticed that the situation on the border was predominantly a threat to the refugees themselves, especially when civil society—activists, NGOs and locals—took action by introducing testimonies from flesh-and-blood to the “migrant crisis” discourse. Slightly more than a third of the respondents thought that time that the situation on the border was a threat to them personally and to their families. Nearly 10 percent of respondents declared that the situation impacted neither the refugees on the border nor they and theirs. While the Polish state was hostile and aggressive, repelling migrants and refugees back to the forests of Belarus, civil society stood up with targeted aid in the borderlands. In January 2022, for the first time, a survey conducted by the non-profit organization OKO.press^5^ saw more than 70 percent of respondents acknowledge the saving of migrants in the Polish forests as something good, even though half a year earlier, half of all respondents had been in favor of push-backs. This shows the power of the governmental crisis narrative interpreting the situation on the border. It is in line with Walby (2022) reflection that civil organizations are able to reduce the complexity of a societal crisis by clearly demonstrating its causes (war in Syria and armed conflicts in neighboring countries) and consequences (individual human tragedies). When the topic dropped out of the news and only activists helping on the border were still visible and did not give up, the societal attitudes changed evoking humanitarian sentiments. However, the cost was a deep fatigue, even burn-outs on the part of the activists and NGOs providing aid on the Belarussian-Polish border. This shows the resistance and how the dominant narratives can be moved somehow. Up to the above point the dominant anti-migrant narrative had seemed unassailable, but as the results of OKO.press's survey in the example discussed here show, such narratives can be questioned and eventually changed by specific actors (cf. Bello, 2022a,b).

The societal dangers can be seen, especially when comparing the reactions of Poles to various refugee crises. The answers to the survey questions discussed further on in this paper show that the acceptance of refugees by Poles is not unconditional, being less the result of a commonly shared idea of helping, but more of the political and cultural narratives about specific crises, which can change quickly according to political interests and needs (cf. IPSOS for More in Common^6^).

With the welcoming and supportive narrative of the Polish government since the escalation of the Russian aggression toward Ukraine started on February 24th 2022, support for Ukraine, its actions and the Ukrainian migrants fleeing to Poland has been high from the onset of this stage of the war. Shortly after the start of the Russian aggression, the Ukrainians fleeing the war received an enthusiastic and empathetic grassroots reception by the Poles. In the March 2022 Ipsos poll for OKO.press, as much as 61 percent of respondents declared that they had taken part in aid actions in some form. Also, the attitude toward Ukrainians staying in Poland at that time was de facto predominately positive by 92 percent respondents^7^. According to the Eurobarometer survey conducted in April 2022^8^, Poland scored highest in the entire EU's in terms of sympathy and solidarity toward Ukraine and refugees from Ukraine. As much as 88 percent of Polish women and men expressed their approval for the reaction of Polish society (formulated in the survey question as “citizens in our country”) to the war and its consequences—this was during a time when the approval rating of Poland's national authorities was 58 percent (Flash Eurobarometer, 2022)^9^.

In September 2022 in the survey by Ipsos for OKO.press, 6 months after the Russian extended invasion of Ukraine, the support for Ukraine in Poland is still substantial but more mixed than in the past months. In Poland, respondents' commitment to support Ukrainian families has fallen from 61 percent in March 2022 to 40 percent in September 2022^10^. Only 40 percent of respondents indicated that they were personally involved in aid provision (as compared to 61 percent in March 2022), and 32 percent had someone in the family who continues to help refugees (as compared to 49 percent in March 2022). Most respondents-−41 percent—had a close friend or acquaintance who supported Ukrainian refugees (as compared to 45 percent in March 2022). This shows an alarming emergence of the societal fatigue syndrome, and it is important for the state to step in with organized and structural aid—all the more so because after 120 days of the war in Ukraine, the Polish government stopped supporting people who provide shelter to refugees. The payment of the benefit of PLN 40 per person per day is now extended only in exceptional circumstances, e.g., in the case of people with disabilities and children up to the age of 12 months. This shows how even for white, European refugees, aid cannot be taken for granted, and also governments may change their minds and withdraw support. This complicates the view that Ukrainians receive unconditional support because they are white—the issue is clearly much more complicated than that^11^.

### 4.2. Danger 2. Othering

The second danger of migrant-related policy narratives, especially those relating to the migrant crisis, consists not only of the *othering* and *labeling* of migrants, but also in the application of double standards in relation to migrants of various origins. According to Appadurai (2006), *othering* seeks to enhance the process of “we-making”, which is by definition short-sighted and limited. It is a by-product of the process of “theys-creating”. Through othering, people build up “predatory identities” (Appadurai, 2006) which social construction and mobilization require othering and therefore helps to build “we-ness”. It also helps establish “our” entitlement to rights. Othering creates a deservingness to be part of a society, or even a nation. It is also about targeted, ill-fated stranger and stigmatized stranger (cf. Goffman, 1968). Othering is exclusionary in nature and creates “insignificant others” (as compared to significant others), and as such helps to contextualize migrants as existing beyond society and to dis-embed migrants from a society. By virtue of othering, migrants are made unintegratable. Othering highlights the modern binaries that are exacerbated by nativist discourses: migrant vs. native, we/us vs. they/them, good migrants vs. bad migrants.

Othering can therefore also be a function of a lack of readiness to offer mutual regard and non-antagonistic coexistence (Collier, 2013) and above all a function of a policy panic (Collier, 2013) where the narrative of othering toward migrants helps to divert the attention of various social groups from recurrent problems. This approaches the territory of a third societal danger, political functionality, which we discuss further on in this article.

The concept of proximization is very compatible with the societal danger of othering due to migrant crisis narratives. Proximization involves the presentation of physically and temporally distant entities, actors or events as “them;” “they” are portrayed as gradual invaders of “our” space, or to put it differently, “they” are conquering “us” (Cap, 2008, 2015, 2017, 2018). Three types of proximization can be distinguished: (1) spatial—“them” as conquering “our” terrain; (2) temporal—deliberately construing an episode as historical momentum which requires immediate preventive and effective measures, such as declaration of a state of emergency; and (3) axiological—portraying “them” as aliens (Cap, 2015).

For instance, the situation of othering and like-us-making is especially visible in Poland, as evidenced by the results of the previously mentioned public opinion poll carried out by Ipsos for More in Common (May 2022)^12^. In response to the question “do migrants and refugees trying to reach Poland *via* the border with Belarus deserve the same assistance as refugees from Ukraine?”, 35 percent said “definitely not”, 25 percent “mostly not”, 21 percent “mostly yes”, and 14 percent “definitely yes”. As interpreted by the non-profit media organization OKO.press, this poll demonstrated the sensitivity of the issue of assistance to refugees and migrants in Poland to political interpretations. The fate of a person seeking help was less important than where they came from; the question of why they were seeking aid in Poland was more important than whether they were indeed in need of aid. In this case, the fact that refugees and migrants were being used by the regime of the Belarusian dictator Alexander Lukashenka to destabilize the situation on the Eastern border of Poland was evidently considered as more important than the tragedies of individual people seeking help from the Polish population (Tomczak, 2022).

In the second round of our expert-stakeholder study under H2020 MIMY research project, we asked respondents from seven European countries what they thought about the different treatment of “different migrants”, as for instance with Ukrainians now in 2022 and Syrians in 2015, seen in several European countries? The vast majority of the stakeholders replied that all migrants who are war refugees should be treated equally, regardless of their country of origin (see Tables 1, 2).

**Table 1:** Stakeholders on the treatment of various groups of migrants^*^.

**Category**	**Policy makers and advisory (*n* = 10)**	**Policy users and advocacy (*n* = 14)**	**Both policy makers and users (*n* = 13)**	**Observers (*n* = 8)**	**Total**
All migrants who are war refugees should be treated equally, regardless of their country of origin	9	14	12	8	43
War refugees should have access to different set of rights/be subject to a different set of integration policies depending on their geographical distance/closeness to the host country	0	1	1	1	3
War refugees should have a different set of rights, benefits and obligations depending on their cultural proximity to the receiving host country.	0	0	1	1	2

^*^Question formulation: What do you think about the different treatment of “different migrants”, as for instance with Ukrainians now in 2022 and Syrians in 2015, seen in several European countries?

Source: H2020 MIMY.

**Table 2:** Stakeholders about Ukrainian refugees and other groups of migrant and their temporary status^*^.

**Category**	**Policy makers and advisory (*n* = 10)**	**Policy users and advocacy (*n* = 14)**	**Both policy makers and users (*n* = 13)**	**Observers (*n* = 8)**	**Total**
Yes	2	2	2	0	6
No	7	12	9	7	35
No opinion	1	0	1	1	3

^*^Question formulation: Should war refugees be treated differently depending on their temporary status (the length of time they are going to be in the country)?

Source: H2020 MIMY.

However, in the open questions, they also added that:

*Migrants should be treated equally within the migrant group. Refugees should be treated equally within the refugee group*. [anonymous]

*But different situations might require different measures*. [anonymous]

*Equal does not mean the same e.g. the number of hours of a language course will be different, the degree of discrimination that should be counteracted with different methods will also be different*. [anonymous]

*Different treatment only breeds racism between different groups in society*. [anonymous]

*It is terribly tough to work with non-European people from war-torn areas and see that society embraces other people because of the principle of closeness*. [anonymous]

The stakeholders were also asked whether war refugees should be treated differently depending on their temporary status, such as the length of time they are going to be in the country. The vast majority of stakeholders said no.

In their qualitative replies, the stakeholders also provided the following comments:

*The problem is due to the existence of two different legal frameworks: temporary protection status or status of applicant for international protection*. [anonymous]

*How can we request integration when we grant temporary residence permits? We are only putting people in a very stressful and uncertain situation and that creates neither conditions nor trust and makes it impossible for people to create a life for themselves in the new country*. [anonymous]

The stakeholders of the H2020 MIMY research project were also asked about structural and relational barriers on the side of both migrants and local populations constraining integration as a reciprocal process including both the migrants and the local population. For this purpose they were presented with a prescribed list of relational barriers developed on the basis of earlier findings from H2020 MIMY research project. Of these, the stakeholders most frequently chose the following relational barriers with regard to migrants: (1) language acquisition (39/45); (2) meaningful contact with members of the receiving society and support (36/45); and (3) local social networks (34/45) and intercultural support and diversity (34/45). The top three relational barriers on the side of local population were (1) intercultural exchange/diversity (36/45); (2) mutual knowledge (34/45); and (3) respect (32/45). In the top three structural barriers to integration on the side of migrants, they indicated: (1) lack of access to resources—jobs, education, housing services (36/45); (2) lack of suitable accommodation—for asylum seekers, lack of privacy in reception centers (32/45); and (3) trauma as a result of experiences in refugee camps, the passage, and/or conflict in the country of origin (30/45) and xenophobia, racism, discrimination and hostility (30/45). In the top three structural barriers on the side of local population they indicated: (1) intercultural exchange/ diversity (36/45); (2) mutual knowledge (34/45); and (3) respect (32/45). Especially the last point on respect is in line with the deliberations of Appadurai (2006) and Collier (2013).

### 4.3. Danger 3. Functionality for political reasons

The third danger of the migrant crisis narrative relates to its functionality for political purposes. Right-wing politicians use migration as a substitutive argument—they consider themselves empowered to categorize migrants as good and bad—which ties in with the previously discussed danger of othering—and use these categories as suits their political convenience, situation, public opinion and electoral support. Migration thus functions as a “whipping boy” for politicians, one that can be blamed for other, often completely unrelated, issues. The political functionality of the migrant crisis narrative often involves highly politicized settings where expert knowledge is contested by “common knowledge” (Lievrouw, 2011) or “laymen's knowledge” (Fischer, 2000), and “fact free politics” are practiced due to the growing activation of the populist sections of society (cf. Scholten and van Nispen, 2015, p. 8).

By overusing migrant crisis narratives in the public sphere, right-wing and populist politicians seek to divert attention from other recurrent social problems, such as access to health systems and the quality of health services, housing problems, taxes and energy costs. Migrant crisis narratives as used by right-wing and populist parties are underpinned by agonistic politics of coexistence, which creates civil distance, tensions, exclusions and conflicts. Politics—and, therefore, policies relating to “migrant crises” is often *ad hoc*, piecemeal, and reactive rather than visionary.

The manner in which right-wing politicians refrain from using migrant crisis narratives also clearly illustrates its functionality. For instance in Scandinavian countries, as showed by Näre et al. (2022), in contrast to the reception of refugees from Middle East, the Ukrainian refugee flow has not been labeled as a “refugee crisis” even though in many receiving countries the number of Ukrainian newcomers exceeded the number of refugees who arrived in 2015–2016. Even right-wing populist parties welcomed Ukrainians in the Nordic countries. For instance, Sweden Democrats have stated that Ukrainians must be helped on a temporary basis and that they should not integrate in Sweden. According to the Finns Party, those fleeing from Ukraine deserve protection, help and assistance because they are Europeans, Christians, and mostly women and children. The image of young men being the main “faces” of the “crisis” in 2015–2016 was reproduced in the media but was not enough scrutinized in public statistics (after Näre et al., 2022), and this image is functionally and manipulatively used by right and far-right politicians.

Another example of the functionality of migrant crisis narratives for political purposes relates to the Polish right-wing coalition government, who used the situation on the Belarussian-Polish border in Autumn 2021 to legitimize its nationalistic policy of a mono-ethnic, Catholic Poland and to cement its own electorate—according to a public opinion survey conducted by Kantar for the center-left daily newspaper Gazeta Wyborcza in November 17–18, 2021, 70 percent of supporters of the ruling Law and Justice party (predominantly men with low education) favored the hostile policy of denying access to the Polish territory for Middle Eastern refugees and migrants and repelling them to the Belarusian territory. In general, 54 percent of respondents supported the actions of the Polish government on the Belarussian border in autumn 2021. The same percentage of respondents also positively rated the idea of building a wall on the border. With regard to the question “do you agree with the following statement: refugees staying on the Polish-Belarusian border must be admitted and allowed to stay in Poland”, 26 percent of the respondents were in favor, and 69 percent against.

As documented by OKO.press, Polish activists noticed how politically functional the migration crisis can be:

“(…) The same politicians who have condemned dozens of other people [Syrian, Eritrean and other Middle Easterners] to starve in the icy forest boast their solidarity with [Ukrainian] refugees. The reserves of great organizations and social empathy have unlocked now, though the humanitarian crisis has lasted since August [2021]. All this enormous help—warm homes and clothes, medical care or universal compassion—were not found by people of a different skin color, fleeing less European wars^13^”. (Tomczak, 2022).

(…) This “manually controlled” asylum policy was very well seen in Afghanistan. The refugees who arrived by plane (and let us remember that the state evacuation was a result of pressure; at first, Afghans were offered humanitarian visas from New Delhi), now find housing and work. But the Afghans, who were freezing and starving in Usnarz, were no longer appealing to the authorities. Although we know that there were people cooperating with Western troops among them^14^. (Tomczak, 2022) [cf. Lopez and Ryan (accepted)].

It is interesting that from the onset of the Russian on-going invasion of Ukraine in February 2022, the Polish government has not directly used a migration crisis narrative when addressing the Ukrainian refugees, while it was the common slogan used with regard to the situation on the Belarussian-Polish border in Autumn 2021; politicians actively commented on the “migration crisis situation” in that region across all media. In 2022 the crisis narrative linked to migration has started appearing, not only in the Polish political space but also the European, when interplayed with inflation crisis and energy crisis. The functionality of the crisis narrative is proportionally equal to the political needs and election calendar, and sometimes a “migration” adjective is instrumentally added to it when this promises to be politically beneficial.

Why is the crisis on the border between Poland and Belarus both defined as a “migration crisis” as well as a “security threat”? It is worth referring here to Bauman's social production of “worse” among “others”? This needs a distinction of criteria that differentiate people. Bauman frames racism as a tool for social engineering (Bauman, 1989). What causes migrants from the Ukrainian border to be allowed accessing Poland, and those from the Belarusian border to be pushed back? Both strategies are inscribed into a policy migrant narrative functionally used by the right-wing ruling government in Poland. It is worth reminding here that nationalist and anti-immigrant attitudes in Poland were presented by people from the lower social classes and the “2015 migration crisis” was among the factors that encouraged them to take part in the 2015 elections. (cf. Ost, 2018; Żuk and Żuk, 2018a).

## 5. Concluding implications for theory and policy

The “migration crisis” narrative has reached a high level of popularity in many European countries. As Vertovec and Wessendorf (2010) show, the perception of a “crisis” was one of the key drivers behind the backlash against multiculturalism across Europe. In various countries, such as the Netherlands, France and the UK, the narrative of a “migration crisis” became linked to rising Euroscepticism connected with the narrative of a lack of national sovereignty. The integration policies of various European countries were also blamed for the crisis.

“A crisis may be contained or it may cascade through the social system; the cascade can be short-lived and minor, or it can be of long duration and major effect. The crisis may be contained (recuperation), have minor effects (intensification), have major effects (transformation) or be total (catastrophe). The outcome depends upon the nature of the social system, on how social systems are connected together and on its level of instability” (Walby, 2022, p. 7). This raises a question how can migration crisis lead to deeper, more lasting social change? If there was a recuperation effect, we would experience *othering* to a very limited extend because all migrants, independent of the type of migration flow, geographical proximity or distance, ethnicity, religion and gender would be embedded into societal inclusion and equality frameworks rather than border, migration and integration regimes and policies. If there was an intensification effect, all the societal dangers discussed in this article—*societal fatigue, othering* and *functionality for political reasons*—would be reinforced and exacerbated, resulting in the “total” crisis that Walby (2022) names a catastrophe. However, societies engage in a great deal of self-balancing activity, aiming for an ideal societal equilibrium—where the state withdraws, a civil society steps in which is, by definition, asymmetric. If the transformation effect took place, migration would be normalized in a society—a new everyday normality would emerge; since migration processes cannot be denied and migrants cannot be just simply thrown away, they are indigenous part of societies. The modern binaries created through othering—such as migrants vs. natives, we/us vs. they/them, good migrants vs. bad migrants—would disappear. We know that these conceptual constructs are Weberian ideal types which can be imputed as based on the findings presented in this article but still remaining ideal societal types.

These ideal types provoke questions, however, about the suitability, sustainability and success of past border, migration and integration policies and the lack thereof. Crises may create a learning opportunity for states and organizations (cf. Kushnir et al., 2020). We do not know what governments have learned from the 2015 crisis, the 2021 Belarus-EU crisis, and the 2022 Ukrainian crisis discussed in this article, but we know that civil society learned a great deal: (1) societal fatigue in a form of a compassion fatigue and aid burnout are real and are caused by the limited involvement of the state; (2) othering is a socially damaging process that causes frustrations, tensions and conflicts; (3) fact-free or fact-silencing or non-fact-scrutinizing politics grows the gap between state and civil society.

Our paper aims to contribute to migration research and sociology in four specific ways. Firstly, we distinguished a policy/government narrative from an individual narrative as discussed by Lopez and Ryan in this Special Issue. The policy narrative exists somewhere between the local, subnational, national and the global levels. The national level is usually constructed by ruling governments, local level is usually constructed by civil society through actions on the ground and the provision of testimonials in the general narrative, while the global level is constructed by international organizations and global traditional and social media. In this article we mostly exploited the space between the national level policy narrative that is functionally created by right-wing and far-right parties and ruling governments, and the local level with the active presence of civil society—NGOs, activists, border area inhabitants and local stakeholders. Through this juxtaposition we identified a gradually growing societal fatigue leading to an aid burnout of civil society, especially when the state withdraws and manipulates migration issues.

Secondly, we identified the factors stimulating migrant crisis narratives. In particular, the *new order of social uncertainties and risks* as initially formulated by Appadurai (2006) played a key role in cataloging these factors. The new order starts with uncertainties and anxieties about stability, existence, state goods and their redistribution. It then proceeds with a partial or complete lack of connection with Weberian predictable bureaucratic and legalized procedures and protocols (e.g., new border crossing protocols, phasing out of migrant integration protocols, and undermining of human rights protocols). This is followed by problems with predictions caused by unexpected occurrences with global impact, such as pandemics and war got identified, followed by lack of security of health and sanitation services. Appadurai also adds a lack of affordable housing for under-waged, young, lower middle class, who are the backbone of a society. We would add to this a new order of uncertainties offered by Appadurai, migration from risks in countries of origin to new risks in Europe which has always been a safe place, a promised land.

Thirdly, we geographically expanded the discussion about migrant crisis narrative (from Western, Southern and Northern Europe) into Central and Eastern Europe. We juxtaposed the situation on the Belarussian-Polish border, where the migrant crisis narrative was purposively overused by right-wing politicians, with the situation on the Ukrainian-Polish border, where the migrant crisis narrative was deliberately not used. Additionally, this paper also discussed voices of resistance as reflected in public opinion surveys and NGOs who can challenge the political narratives (cf. Bello, 2022a,b).

Fourthly, we identified the societal dangers of the overuse and deliberate non-use of migrant crisis narrative, which causes societal fatigue, othering and political functionality, all phenomena that shake societies.

As we said at the beginning of the article, processes of migration are here to stay, and will be serial, not episodic. Migration crisis narratives as social constructs are very effectively used and abused by politicians. It is easier for politicians to talk about “a general migrant problem” than of the system or the condition of a country under their rule. The unintended social consequences of these political practices relate to the critical role of the civil society that is at the core of the matter.

## Data availability statement

The raw data supporting the arguments of this article will be available according to the rules of the Grant Agreement of H2020 MIMY research project and scrutinized by the Research Ethics Committee.

## Ethics statement

The studies involving human participants were reviewed and approved by Research Ethics Committee, Kozminski University, Warsaw, Poland. The patients/participants provided their written informed consent to participate in this study.

## Author contributions

The author confirms being the sole contributor of this work and has approved it for publication.

## Footnotes

^1^ https://cordis.europa.eu/project/id/870700

^2^Germany (*n* = 12); Italy (*n* = 17); Luxembourg (*n* = 8); Poland (*n* = 17); Romania (*n* = 17); Sweden (*n* = 17); UK (*n* = 15); Others (EU, Hungary, no data) (*n* = 11).

^3^Rather than a general term we need to differentiate between visible (undesired) and invisible (desired) migrants—see Mulholland and Ryan (2022) on how migrants may experience different levels of visibility and invisibility and how that can change, which can have unsettling consequences.

^4^This is important due to the fact that the majority of migrants in 2015-16 entered the EU *via* the sea border of Italy.

^5^https://oko.press/uchodzcy-gorszego-sortu/ IPSOS survey for More in Common, telephone survey (CATI) on 4–7 May 2022, sample of 1,000 people, representative of adult Poles.

^6^https://oko.press/uchodzcy-gorszego-sortu/ IPSOS survey for More in Common, telephone survey (CATI) on 4–7 May 2022, sample of 1,000 people, representative of adult Poles.

^7^ https://oko.press/uchodzcy-gorszego-sortu/

^8^ https://ec.europa.eu/migrant-integration/library-document/eurobarometer-europeans-approve-eus-response-war-ukraine_en

^9^Survey conducted by Ipsos European Public Affairs at the request of the European Commission, Directorate-General for Communication between 13-20 April across EU Member States.

^10^ https://oko.press/fala-pomocy-ukrainskim-uchodzcom-opada-dowodzi-sondaz-oko-press-wladza-pis-zawiodla/

^11^See: Polish Act of March 12, 2022 on helping Ukrainian citizens in connection with an armed conflict in the territory of that country (In Polish: USTAWA z dnia 12 marca 2022 r.o pomocy obywatelom Ukrainy w zwiazku z konfliktem zbrojnym na terytorium tego państwa).

^12^ https://oko.press/uchodzcy-gorszego-sortu/
